# Cluster-Like Headache Secondary to Sphenoid Sinus Mucocele

**DOI:** 10.1155/2018/5850286

**Published:** 2018-12-05

**Authors:** Mariana Branco, Rita Rodrigues, Marta Lopes, Luís Ruano

**Affiliations:** Neurology Department, Hospital São Sebastião, Entre Douro e Vouga Hospital Centre, Rua Dr. Cândido de Pinho, 4520-211 Santa Maria da Feira, Portugal

## Abstract

**Background:**

The great majority of cases of cluster headache (CH) are primary, but there are several reported cases of CH secondary to underlying structural lesions. The identification of these lesions is crucial for the achievement of an effective treatment and favorable outcome, although the determination of a cause-effect relationship between the two entities may be challenging.

**Case Report:**

We present the first case of CH secondary to sphenoid sinus mucocele.

**Discussion:**

This case reinforces the need to perform neuroimaging studies in CH patients in order to identify lesions that can constitute its cause, especially if atypical features are present. Activation of the trigeminovascular system due to direct contact between the lesion and the trigeminal nerve or by local edema and inflammation possibly plays a role in the pathophysiology of this CH secondary to sphenoid sinus mucocele.

## 1. Introduction

Although the great majority of cases of cluster headache (CH) are primary, a small number of patients present an underlying structural lesion [1]. The diseases most often associated with cluster-like headache are pituitary adenoma, vascular loops, fungal sinusitis, or tumors, being frequently difficult to determine a cause-effect relationship. Suggestive spatial location, temporal link, and relief of symptoms after treatment of the associated lesion are arguments in favor of a causal association [2]. The true prevalence of such cases is unknown, as there are no prospective population-based studies of CH with systematic cerebral imaging [1]. Recognizing these underlying pathologies is of crucial importance, as they can influence treatment and outcome [3].

Sphenoid sinus mucocele is a rare condition, comprising 1–2% of all paranasal sinuses mucoceles. Despite its benignity, it may displace or compress several vital contiguous neurological and vascular structures: cranial nerves II to VI, cavernous sinus, carotid artery, sphenopalatine artery and nerve, pterygoid canal and nerve, dura and pituitary gland [4].

## 2. Case Report

**Figure 1:**
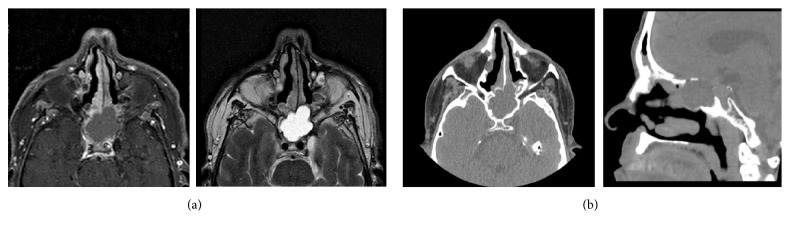
Neuroimaging of the sphenoid sinus mucocele. (a) Paranasal sinus MRI revealing molding of the medial wall of left cavernous sinus by the sphenoid mass. (b) Paranasal sinus CT scan showing sclerosis and interruption of the roof and posterior wall of left sphenoid hemisinus.

A 62-year-old man presented at the Neurology consultation with a six-week history of a severe, strictly left orbitotemporal headache, with a frequency of three attacks per week, occasionally more than one at the same day. Most of them occurred in the first half of the night, waking him up, and lasted between thirty minutes and one hour. He used to take ibuprofen as acute treatment, with unsatisfactory response, since he did not notice a significant difference between treated and untreated attacks in terms of duration and pain intensity. To relief the pain, he used to open the window to get some fresh air. The headache was always associated with ipsilateral conjunctival injection and lacrimation. Pain triggers were not identified by the patient. He had no personal or familial history of headaches. His medical history was remarkable for hypertension and asthma, with a past surgical history including septoplasty and bilateral middle turbinectomy and uncinectomy due to nasal respiratory insufficiency. By the time of medical evaluation he was asymptomatic and neurological exploration was unremarkable. The clinical picture was suggestive of a CH and the patient was medicated with verapamil 120 mg daily. A MRI scan was performed, which revealed a sphenoid sinus mucocele, without secure expansion of the sinus. Two weeks later the patient came to the Emergency Department with complaints of horizontal diplopia that he noted when he woke up in that morning. He maintained the headache attacks, with similar characteristics, despite prophylactic therapy. Neurological examination revealed left eye adduction palsy and ptosis. A brain CT scan was performed and excluded lesions other than the mucocele. Paranasal sinus MRI revealed molding of the medial wall of left cavernous sinus by the sphenoid mass (Figure 1(a)). A paranasal sinus CT scan was also performed to allow for a better characterization of the lesion, showing sclerosis and interruption of the roof and posterior wall of the left sphenoid hemisinus (Figure 1(b)). The patient was submitted to surgical drainage of the mucocele by transnasal-transphenoidal approach, with complete resolution of the adduction impairment, persisting a mild left eye ptosis. After the surgery the attacks stopped, and in the six-month follow-up he reported no further attacks.

## 3. Discussion

There are several reported cases of CH in the context of a structural lesion [1]. However, to our best knowledge, no cases of CH secondary to sphenoid mucocele have ever been reported. A previous case of oculomotor nerve palsy associated with trigeminal-autonomic features secondary to sphenoid sinus mucocele was reported, but without headache [5]. Another previous report recently described the first CH secondary to maxillary sinus mucocele [6].

The clinical picture of our patient fulfilled the diagnostic criteria of cluster headache [7]. The age of onset was atypical, but primary CH has been reported in all age groups [8]. The patient did not notice a significant response to ibuprofen, which was not surprising, since CH attacks are short-lived and, therefore, oral routes of administration are not expected to be effective (ibuprofen onset of action is usually thirty minutes to one hour after administration [9], corresponding to the duration of the untreated attacks). Concerning prophylactic therapy, the patient did not present response to verapamil, but the administered dose (40 mg, three times a day) was the lowest effective in preventing this type of headache, and higher doses are usually required [10]. However, for safety concerns, we performed a slow dose titration, as our patient was older than the majority of cluster headache patients and verapamil has the risk of cardiac side effects [11]. Therefore, when he went to the Emergency Department, he was still on this initiation dose.

The finding of a sphenoid sinus mucocele in the first MRI raised the suspicion of a secondary CH, but we could not establish an unequivocal cause-effect relationship between the two entities at that time. The development of an oculomotor palsy short after the onset of the headache, which was a direct complication of the sphenoid sinus lesion, implied an urgent surgical intervention, after which a sustained remission of the pain was observed, allowing the determination of the mucocele as the cause of the CH.

The mechanism of pain and development of trigeminal-autonomic symptoms in CH is still controversial, but it is believed that the trigeminovascular system may play a role in its pathophysiology [2]. In our patient's case, we hypothesize that the pain and trigeminal-autonomic features may be due to direct contact between the lesion and the trigeminal nerve or secondary to local edema and inflammation.

This case shows that CH may be one of the multiple neurological manifestations of sphenoid sinus mucocele and reinforces the importance of neuroimaging studies in trigeminal-autonomic headaches. We suggest that, in the concomitant and ipsilaterally presence of these two entities, a cause-effect relationship should be strongly considered and surgical removal of the lesion should be performed as soon as possible, as it can be the only way to treat this disabling type of pain and prevent other serious neurological complications.
